# Social Robots to Test Flexibility of Human Social Cognition

**DOI:** 10.1007/s12369-020-00674-5

**Published:** 2020-07-10

**Authors:** Agnieszka Wykowska

**Affiliations:** Social Cognition for Human–Robot Interaction Unit, Istituto Italiano di Tecnologia, Genova, Italy

**Keywords:** Human social cognition, Social robotics, Experimental psychology, Cognitive neuroscience, Human–robot interaction

## Abstract

As the field of social robotics has been dynamically growing and expanding over various areas of research and application, in which robots can be of assistance and companionship for humans, this paper offers a different perspective on a role that social robots can also play, namely the role of informing us about flexibility of human mechanisms of social cognition. The paper focuses on studies in which robots have been used as a new type of “stimuli” in psychological experiments to examine whether similar mechanisms of social cognition would be activated in interaction with a robot, as would be elicited in interaction with another human. Analysing studies in which a direct comparison has been made between a robot and a human agent, the paper examines whether for robot agents, the brain re-uses the same mechanisms that have been developed for interaction with other humans in terms of perception, action representation, attention and higher-order social cognition. Based on this analysis, the paper concludes that the human socio-cognitive mechanisms, in adult brains, are sufficiently flexible to be re-used for robotic agents, at least for those that have some level of resemblance to humans.

## Introduction

The field of social robotics has been growing with increasing momentum. The International Journal of Social Robotics—which has recently celebrated its 10th anniversary—has been covering a broad spectrum of research and application areas of social robots, which are being developed to assist humans not only in mundane daily activities but also in healthcare, elderly- and childcare as well as educational activities. Quick overview of the papers published over the years in the area of social robotics across various journals and conferences illustrates that a large amount of effort in social robotics has been dedicated to healthcare (e.g., [1–6] for an overview see [7–9]), elderly care [10–13], therapeutical interventions for children [14–23], or educational applications [24–29].

Therefore, one can conclude, based on this collected body of literature, that social robots serve the purpose of being humans’ assistants and companions for not only in healthcare applications but also in daily lives fostering collaboration in the workplace [30] or assistance at homes, airports and supermarkets [31–33].

This paper, however, provides a different perspective on the role of social robots, as it elucidates that social robots can be scientific tools to examine human social cognition system, and its flexibility in particular. The question of interest is whether similar mechanisms of social cognition are elicited by social robots as those elicited by other humans. This paper focuses on examples of socio-cognitive mechanisms that have been studied with robot and human agent stimuli/interaction partners, and the socio-cognitive mechanisms have been directly compared between the two types of agents. The mechanisms this paper addresses are: the perceptual system, action representation system, attention and higher-order cognition.

## Encompassing Robots in the Perceptual Apparatus Developed for Natural Agents

Several authors examined the impact of appearance of agents (often presented as either avatars or virtual agents) on perceptual processes. For example [34] investigated a spectrum of virtual characters ranging from very artificial-looking to natural human-like, all presented in a running movement. The movement was either based on motion capture data from actual humans performing the movement or was constructed through a “key-framing” technique by an animator. Participants’ task was to judge whether the presented movement is biological or artificial, and the key question was whether appearance of the agent would influence that judgment. The results showed that participants had quite good sensitivity in detecting biological motion, and this sensitivity was modulated by appearance only in case of significantly reduced in content stimuli (dot characters vs. full-body characters). However, there was not much difference in sensitivity to biological motion across robot and human characters. This shows that the perceptual system which is sensitive to biological motion might not respond selectively to human-like appearance.

Similarly [35] showed that the action perception system is not selective to biological motion or appearance, suggesting its “flexibility”, in the sense that it responds similarly to both the natural and artificial agents. Importantly, however, some regions of the system (bilateral anterior intraparietal sulcus specifically) responded more pronouncedly to the condition in which an agent had a very human-like appearance (an android) but mechanistic behaviour, suggesting that those regions are a neural marker of the uncanny valley phenomenon [36].

However, as the stimuli in these studies were 2D, presented on the screen, it remains to be examined whether appearance would not affect perception of biological motion when the observed character is actually a physically present embodied agent.

In the context of perceptual system for emotional expressions, Chaminade and colleagues [37] found that participants rate intensity of expressed emotions higher for human face stimuli, as compared to robot face stimuli, when the emotions are negative (anger and disgust specifically). Interestingly, at the neural level, brain regions related to perceptual processing showed higher activation for robot stimuli, compared to human stimuli, but areas implicated in emotional resonance showed reduced activity for the robots. The authors conclude that robots with highly mechanical features might not induce the same degree of resonance as other human conspecifics. Due to unfamiliarity with robots, the perceptual levels of processing might have required engagement of additional resources in order to for example, recognize a robot “face” as a face. Interestingly, as there was no difference between robot and human stimuli in processing of gestures in the area typically responsible for gesture recognition (right superior temporal gyrus), this shows the flexibility of human perception for reusing the same system for human and artificial agents, in terms of gesture recognition.

## Re-using Action Perception System for Representing and Resonating with Robot’s Movements

Researchers addressed the question of whether the neural system underlying action representation when observing other humans is activated also when observing the actions of robots. Specifically, the crucial question to ask is the importance of human-like motion appearance on motor resonance [38, 39]. Some authors showed a diminished effect of motor resonance during observation or imitation of robot actions [40, 41]. Specifically, Kilner et al. [40] showed that motor interference effect occurring during observation of actions that are incongruent to the concurrently performed actions can be observed when participants perform the action together with another human, but not with a robotic agent. Interestingly, when an industrial robotic arm has been replaced by a more human-like humanoid robot [42], the interference effect was similar for the humanoid and the human agent conditions, an effect observed also in [43]. Similarly [41] showed interference effects in an imitation task for both human and robotic hand stimuli. However, the effect observed in their study was more pronounced for human than for robot stimuli. It is plausible, though, that the slightly smaller effect for the robot condition was due to that the robot hand stimuli were presented on a screen, thus lacking natural embodied presence that actual robots offer when placed in front of participants. However, another study [44], followed up this line of research and demonstrated (with video stimuli presented on a computer screen) that human-like joint configuration is the critical factor for interference effects to occur.

In a different type of paradigm, Wykowska and colleagues [45] showed that a system for action representation, which influences attentional selection of perceptual features, is similarly activated by robot-like arm stimuli (depicting pointing and grasping actions) as by human-like arms representing the same actions. This speaks in favour of re-using the action-perception system developed for representing actions of other humans for representing also actions of artificial (human-like) agents.

In a study using functional magnetic resonance imaging (fMRI), Gazzola and colleagues [46] showed that the mirror neuron system responded similarly to robot and human actions, as long as same actions were not repeated over many trials. This certainly shows that the motor resonance system is sufficiently flexible to respond not only to natural but also artificial agents. However, [47] reported results seemingly at odds with the previous findings. In an experimental design where dynamic stimuli (videos) of human and robot-like agents were presented to participants in a smooth, fluid, human-like dance motion or displaying a rigid robot-like movements, the action observation system was actually more strongly activated for the robot-like movements, compared to the human-like fluid motion, independent of the agent type. Similarly to the findings in [34], this suggests that the human perceptual and action observations systems are more sensitive to movement characteristics than to the form of the agents itself. Interestingly, the study in [47] shows—in contrast to previous interpretations of the function of the action observation system [48]—that the system is not necessarily selective to actions that are familiar to the observer, or that are closer to the observers’ motor repertoire. On the contrary, it might show a non-linear response pattern, with highest activation for extremely familiar and non-familiar movements, and lower activation for movements occupying the space in-between the two extremes.

Another question related to flexibility of action representation system is whether humans are capable of understanding robot’s affordances [49]. As reviewed in [50], some studies showed that humans are capable of perceiving robot’s affordances with respect to moving through an aperture of a certain width [51]. However, as interestingly pointed out in [50], it remains to be examined whether robots with motor repertoire different from that of humans would still allow for correct attribution of affordances. Human-like shape (implying human-like motor repertoire) might be an important factor for the action perception system, a suggestion in line with the findings in [42].

In sum, the state-of-the-art evidence collected in studies focusing on motor resonance or action representation in general show that this system is flexible enough to respond not only to natural conspecifics but also to other artificial agents, as long as some resemblance to humans is preserved.

## Triggering Attentional Orienting by Robot’s Social Signals

Another large body of research has been dedicated to attentional processes in relation to stimuli depicting natural and artificial agents. Specifically, gaze-induced joint attention has been widely investigated. Joint attention is one of the most fundamental mechanisms of social cognition [52]. In fact, when individuals suffer from deficiencies in the ability to engage with others in joint attention, this affects other, multifaceted and higher-order mechanisms of social cognition [52]. Joint attention is a mechanism that allows two individuals to attend the same object or event in the environment [53, 54]. It is an evolutionarily adaptive mechanism [55–57], as attending where others attend can inform us about behaviourally relevant events in the environment, as well as the others’ intentions, action plans and successive action steps [58–61]. Joint attention is often established by directional gaze [52, 57] or other communicative gestures such as pointing [62–65]. In the context of this paper, it is important to address the question of whether similar joint attention mechanisms are evoked by robot agents, as in the case of human stimuli.

This question was examined in [66] with a gaze cueing paradigm [67, 68]. The gaze cueing paradigm is a modified Posner protocol [69] which is typically used to test attentional orienting in response to a directional cue. In a standard Posner paradigm, participants are asked to detect or discriminate a target presented in one (usually lateral) side of the computer screen. A centrally presented directional cue (for example, an arrow) orients attention to one of the sides (either the same side where the target will later appear, or towards a different location. The typical pattern of results shows that participants are better in detecting targets that are presented at cued, compared to uncued, locations, as their attention has been focused on the cued location prior to target presentation. Similar logic has been used for eye-like stimuli, which can serve as directional cues, orienting attention to a specific location. Gaze-related attention orienting (gaze cueing effects) have been observed for schematic face stimuli [67] and also for pictures of human faces [68].

Wiese and colleagues [66] presented participants with robot and human face stimuli. The results showed larger gaze cueing effects for human faces, relative to robot faces. However, this effect was not so much dependent on the actual appearance (robot vs. human) but rather on the belief that participants held regarding who actually “controls” the eye movements (a human agent or a pre-programmed algorithm). This initial behavioural effect was then paralleled by event-related potentials (ERPs) of the electroencephalogram (EEG) signal related to attentional processing [70] and fMRI results [71] indicating that the critical difference in observed in behaviour is mirrored by differential pattern of activation in the bilateral anterior temporo-parietal junction (TPJ), a region typically involved in attentional re-orienting as well as mentalizing [71, 72].

However, it might be that when a robot is more human-like in appearance than the robot used in [70–72], the gaze cueing effects would be elicited to the same degree as in the case of human agents. This is suggested by the series of studies of Kompatsiari and colleagues [73, 74]. In those studies, however, there was no direct comparison with the human, and thus it is not clear whether the observed gaze cueing effects elicited by a robot agent were of the same magnitude as they would be for human agents.

Admoni and colleagues [75] compared the gaze cueing effects across various agent types (pictures of a human face, schematic face drawings, pictures of a Zeno robot (Hanson Robotics^1^), and pictures of Keepon, a robot developed by Hideki Kozima^2^) in a counter-predictive cueing procedure. The results showed no indication of reflexive orienting to directional cues from robot face stimuli. Interestingly, however, there was also no clear indication of reflexive orienting towards human gaze. This might be due to insufficient statistical power in the experimental design (for a similar argument, see [76]).

One interesting question that needs to be asked is to what extent human-likeness of appearance and/or behaviour of a robot elicits attentional orienting mechanisms. Abubshait and Wiese [77] addressed this question in a gaze-cueing paradigm in which both appearance and behaviour were manipulated. The authors presented to participants photographs of human and robot faces which could cue target location with either 50% probability (random condition) or with 80% probability (reliable condition). The results showed that the gaze cueing effects depended on reliability of behaviour, but not on the type of agent. Similarly, Martini and colleagues [78] examined the impact of appearance, showing an inverted-u shape of magnitude of the gaze cueing effect, in relation to human-like appearance. The authors used a morphing technique to present to participants human and robot faces with different degrees of human-likeness. The results showed that morphs that we were more in the fuzzy area in-between human and robot appearance elicited reflexive attentional-orienting response (the cueing procedure was counter-predictive), while 100% human and 100% robot faces induced reverse gaze cueing effects. Results of both studies show that robot and human faces are capable of evoking similar attentional mechanisms of the human brain. In a similar vein, Chaminade and Okka [79] showed that there was no difference in the head-cueing effect between a human and robot agent (NAO^3^), suggesting that the attentional orienting system that is used for responding to directional cues provided by other humans is also used for artificial agents, if the cue is salient enough.

However, and interestingly for the purposes of this paper, Okumura and colleagues [80] examined the developmental factor in gaze-related attentional orienting towards human and robot gaze. The question of interest was whether young children (toddlers) develop joint attention with robots as early as joint attention with humans. The results showed that both 10-month old toddlers and 12-month old children follow the gaze of robots and humans to the same extent. However, only 12-month old children showed anticipatory effects related to an object presented in the cued location, and only for the human faces. The authors argue that this effect is related to expectations and predictions of the upcoming object presentation. An alternative explanation of a sustaining effect of human gaze on attentional focus has been ruled out by the design, which included a fixation point in the middle of the screen reorienting the gaze of children to the center, before presenting anticipatory placeholders in which a target object would later appear, and anticipatory gaze would be measured. The authors interpret this pattern of results in the context of possible difference in communicative content expected in human gaze, but not in robot gaze. This would be in line with the results reported in [66] and in [70] where it has been shown that the belief regarding the agency (human vs. pre-programmed robot) underlying the observed behaviour, independent of the actual appearance, modulates the magnitude of gaze cueing effects. This also suggests the impact of assumed communicative content on engagement in joint attention. Okumura and colleagues [80] discuss two additional potential alternative explanations of their effects: one related to higher degree of experience and exposure to human faces, as compared to robots, and the other being learned associations between human gaze and a subsequent appearance of an interesting event in the environment.

**Fig. 1:**
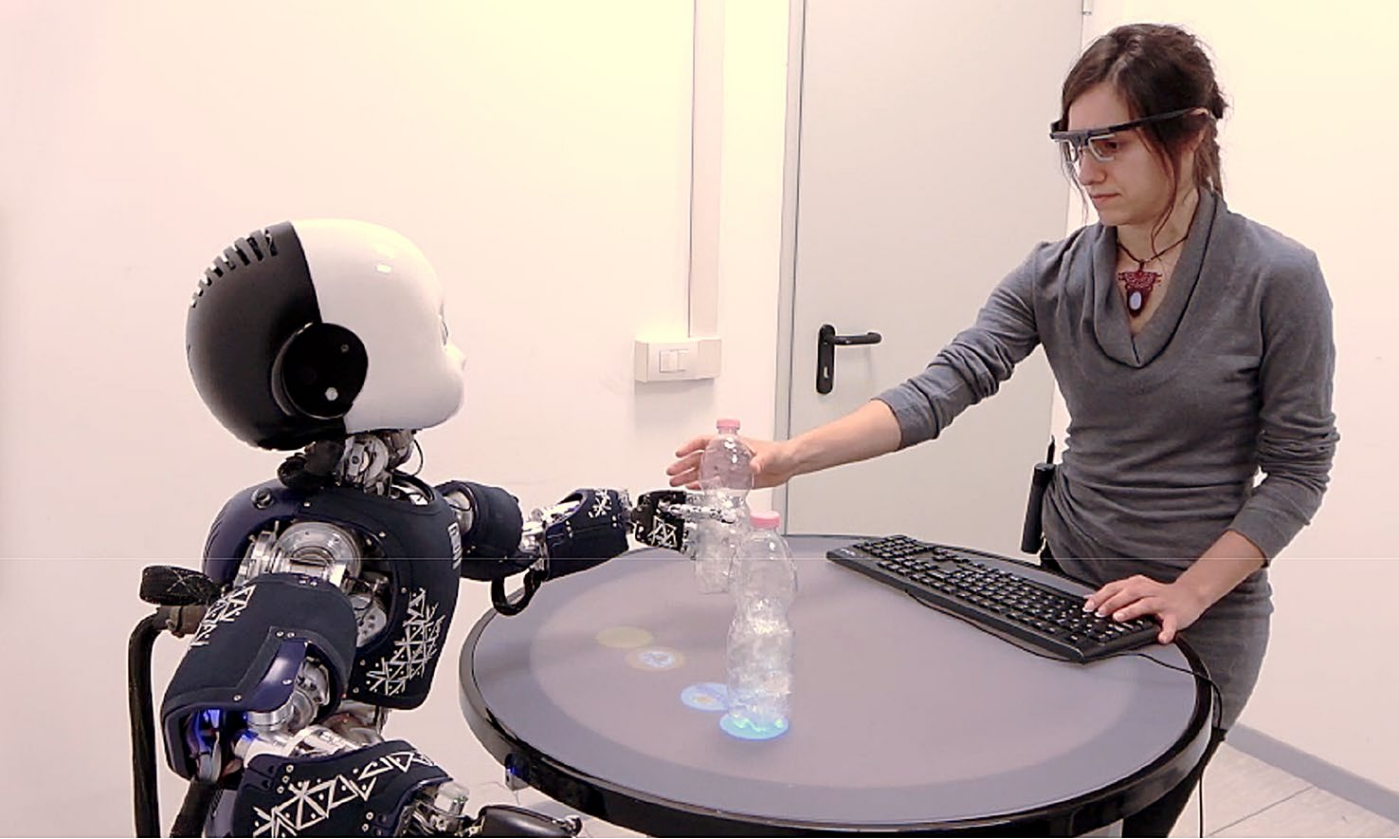
Experimental setup of [60] in which a gaze cueing protocol is embedded in action sequences. The participant (right) is wearing a mobile eye-tracking system allowing for measuring eye movement patterns during the interactive task

In either case, it seems that the human attention system is flexible enough to respond to directional cues provided not only by conspecifics, but also by artificial robot agents. However, this flexibility needs some time to develop, as shown in [80]. One interesting question remaining for future research is whether similar gaze cueing effects occur for human and robot interaction partners in more naturalistic interactive scenarios. Perez-Osorio and colleagues examined gaze cueing effects with human-agent stimuli on the screen [60, 61] and showed that the effects are modulated by action expectations of the observer. In a follow-up study [81], the paradigm was implemented in an interactive protocol with the embodied iCub [82] robot (Fig. 1), and the effect was replicated. However, a direct comparison between an embodied robot and another human co-agent is still missing for drawing definite conclusions.

## The Cognitive System for Reasoning About Others’ Motives Underlying Behaviour

A crucial higher-order mechanism of social cognition is mentalizing, namely tracking others’ mental states [83]. This is one of the key mechanisms allowing predicting and explaining other people’s behaviour. In order to involve in a mentalizing process, one needs to adopt the intentional stance [84], namely one needs to treat the agent they are referring to as an intentional system, capable of having mental states. Several researchers have addressed the question of whether humans activate the mechanisms of mentalising towards robots, as much as they do towards other humans. For example [85] extended a previous paradigm [86] and examined adoption of the intentional stance towards a robot, a computer program and a human agent in an fMRI study. The results showed that at the neural level, human partners uniquely activate mentalizing areas, which are not elicited during interaction with an artificial agent. This might suggest that the mechanisms involved in mentalizing processes are special for social interactions with other humans, and do not necessarily extend to interactions with artificial agents such as robots. Similarly, Levin et al. [87] examined whether participants would attribute human-like mental processes to a robot or a computer in a task with scenarios with open endings. The results showed that intentional ascriptions were significantly more frequent towards human agents than towards a computer or a robot. Also Marchesi and colleagues [88], with a novel tool, the Intentional Stance Questionnaire, showed that participants were more likely to give mentalistic explanations to observed behaviour of a human agent, relative to a robot for whom the mechanistic descriptions were more frequent. However, even though participants were more likely to use mechanistic terms in describing robot behaviours, the mentalistic explanations for robot behaviour were not uncommon. In contrast, Thellman et al. [89], as well as de Graaf and Malle [88], showed that people actually use similar descriptions for robot behavior as they would use for humans, specifically regarding ascription of intentionality.

The fact that humans might (in some cases) ascribe intentionality to robots, and/or explain and predict their behaviour with reference to mental states shows that our cognitive system uses the tools it has developed throughout the history of humankind to accommodate for new types of situations, such as social interactions with artificial agents. This would potentially show flexibility of the cognitive system, whose mechanisms that have been developed for natural interactions with conspecifics and other beings of natural kind seem to be hijacked by other types of agents, those that do not belong to the natural order. However, the existing body of literature related to attribution of mental states to robots provides a mixed picture. It seems that in some cases the mentalizing system is also used for artificial agents, but it is not always the case. It remains to be answered with future research what are the crucial conditions for attribution of mental states to robots. In the literature reviewed here, it might be that the different ways of operationalising intentional stance yielded different results. Indeed, operationalising a high-level philosophical concept, such as intentional stance [84], is quite challenging. For example, in [85], the intentional stance was operationalised in form of an interactive game of rock-paper-scissors where the participants were asked to determine whether they thought they were playing against a computer programme or another human. In this case, the dependent variable was differential activation in the social brain areas that could be interpreted as related to adoption of intentional stance, similarly to [86]. On the other hand, in [88, 89], participants observed static pictures and their task was to choose descriptions of the observed behaviours of either human or robot agents. The dependent variable was subjective ratings regarding mentalistic or mechanistic descriptions of behaviours. In [90] participants read text descriptions of robot or human behaviours and they were asked to provide verbal written explanation of the behaviour. Thus, the discrepancy between results can potentially result from the difference in paradigms (interactive [85] vs. passive observation or reading written text [88–90]; judgment of behaviour of an actually presented agent [88, 89] vs. judgment of agency based on the observed behaviour as in [85]), difference in the adopted measures (brain activity [85] vs. subjective judgments [88–90] as well as type of presented agents (iCub robot [88] vs. Pepper [89]). Finally, the type of body postures depicted in the visual stimuli [88, 89] and context might have also played a role. In order to understand the discrepancy between results and potential factors contributing to the likelihood of adopting the intentional stance towards robots, it would be desirable to develop a common unified way of operationalising certain concepts, and common way of measuring them, and preferably studies performed across various robot platforms to test whether the obtained effects are generalizable across a variety of robot embodiments. This would be an important approach not only in the case of addressing socio-cognitive mechanisms such as mentalising but also a general suggestion for measuring human cognitive mechanisms in the context of social robotics research and human–robot interaction overall.

## Concluding Remarks

In this paper, I aimed to show that social robots – apart from playing the role of assistants and companions in various domains of human life—can also inform us about the flexibility of the human socio-cognitive system, which, in many cases, is apparently capable of incorporating artificial agents—at least those that are to some extent similar in appearance to humans—into the social sphere, by reusing the same mechanism as it would use in interactions with other conspecifics. However, this flexibility might need some time to develop, and some socio-cognitive mechanisms, such as application of social norms, or in some cases mentalising, might be uniquely specific for interactions with other humans. Moreover, in order to draw more generalisable conclusions, this field of research would benefit from replicating studies with unified experimental protocols addressing a cognitive mechanism in question across various robot platforms to test whether the observed effects are not due to a particular robot shape. Despite these limitations of the present state-of-the-art, the literature reviewed in this paper shows how wide is the application and scientific contribution of social robotics, extending beyond application areas to more theoretical and fundamental questions related to human social cognition. Therefore, as the social robotics field is largely multidisciplinary and with broad potential contributions to applied and fundamental science, the International Journal of Social Robotics shall continue to blossom with a wide variety of topics related to research and application of robots for society.

## Footnotes

1 https://www.hansonrobotics.com/zeno/.

2 https://beatbots.net.

3 https://www.softbankrobotics.com/emea/en/nao.
